# Pulmonary haemodynamics and right heart function during exercise at high versus low altitude in patients with pulmonary vascular disease: a randomised crossover trial

**DOI:** 10.1136/heartjnl-2024-325605

**Published:** 2025-05-22

**Authors:** Julian Müller, Laura Mayer, Simon Raphael Schneider, Meret Bauer, Michael Furian, Konrad E Bloch, Esther I Schwarz, Mona Lichtblau, Ulrich Silvia

**Affiliations:** 1 Department of Pulmonology, University Hospital Zurich, Zürich, Switzerland; 2 University of Zurich, Zürich, Switzerland

**Keywords:** pulmonary vascular disease, echocardiography, pulmonary arterial hypertension

## Abstract

**Background:**

Patients with pulmonary arterial hypertension or chronic thromboembolic pulmonary hypertension (PAH/CTEPH) may experience physiological stress at high altitude. We investigated pulmonary haemodynamics and right heart function during incremental (IET) and constant work-rate exercise tests (CWRET) at high (2500 m) vs low altitude (470 m).

**Methods:**

In this randomised crossover trial, patients with stable PAH/CTEPH without resting hypoxaemia performed IET and CWRET at both altitudes. Systolic pulmonary arterial pressure (sPAP) and right ventricular (RV) arterial coupling (tricuspid annular plane systolic excursion/sPAP) were assessed by echocardiography.

**Results:**

Among 27 patients (44% women, 61±14 years), sPAP was higher at rest at 2500 m vs 470 m (mean difference: 14 mm Hg, 95% CI 7 to 23), but increased linearly during exercise with similar slopes at each altitude (7.9 vs 9.7 mm Hg/min, respectively). RV arterial coupling was lower at high altitude at rest (difference: −0.13 mm/mm Hg, 95% CI −0.26 to −0.04) but decreased comparably during exercise. During CWRET, sPAP rose steeply in the first 3 min, plateauing thereafter, with no altitude-dependent differences in pressure-flow slope. Oxygen delivery was reduced at high altitude.

**Conclusion:**

Despite higher baseline sPAP and reduced RV coupling at rest, exercise-induced haemodynamic changes were similar at both high and low altitudes, suggesting short-term altitude exposure does not exacerbate cardiopulmonary stress during exercise in stable PAH/CTEPH. The exercise protocol (IET vs CWRET) alters haemodynamic trajectories more than altitude.

**Trial registration number:**

NCT05107700.

**WHAT IS ALREADY KNOWN ON THIS TOPIC**

- Right ventricular function influences outcomes in pulmonary vascular disease (PVD), but exercise responses at high altitude are unclear.

**WHAT THIS STUDY ADDS**

- Randomised controlled trial showing similar exercise haemodynamic patterns at 2500 m vs 470 m, despite higher baseline systolic pulmonary arterial pressure/right ventricular stress at altitude.
- Exercise protocol (incremental vs constant work-rate) alters haemodynamic trajectories more than altitude.

**HOW THIS STUDY MIGHT AFFECT RESEARCH, PRACTICE OR POLICY**

- Short-term altitude exposure may be safe for patients with stable PVD, but individualised testing is advised.

## Introduction

 In patients with pulmonary vascular disease (PVD), hereafter used as a joint term for pulmonary arterial hypertension (PAH) and distal chronic thromboembolic pulmonary hypertension (CTEPH), the right ventricular (RV) structure and function mainly determine symptoms and outcome.^1^ A key parameter for RV contractile function in response to increased afterload is the RV-arterial coupling.^2^ The tricuspid annular plane systolic excursion/systolic pulmonary artery pressure (TAPSE/sPAP) ratio offers a validated, non-invasive way to estimate the RV arterial coupling by echocardiography.^3^ At rest, a TAPSE/sPAP ratio ≤0.32 mm/mm Hg is a predictive risk factor for all-cause mortality in patients with PVD.^4 5^ However, especially during exercise, when the RV needs to preserve its coupling in response to increased afterload, this parameter might be of great relevance. At high altitude, hypoxaemia leads to hypoxic pulmonary vasoconstriction, exposing the RV to a higher afterload.^6 7^ The degree of RV afterload can be assessed by echocardiography with the pressure-flow relationship—the total pulmonary resistance (TPR) calculated as sPAP/cardiac output (CO) ratio. During exercise, Δ sPAP/Δ CO, the pressure-flow slope was additionally found to be a predictor for transplant-free survival in patients with PVD.^8^ We have recently shown that peak exercise capacity during incremental cycling exercise tests (IET) was 11% lower at 2500 m compared with 460 m of altitude in 27 patients with stable PVD. This reduction was associated with lower blood oxygen content and peak VO_2_; however, perceived dyspnoea was unchanged.^9^ Although cycling exercise in these predominantly low-risk patients was well tolerated, the contribution of the RV function and oxygen delivery to impaired exercise capacity at high altitude, and whether it differs between various exercise protocols, remains unclear.

The aim of this project was therefore to investigate heart function using right heart-focused echocardiography during IET and constant work-rate exercise tests (CWRET) at 2500 m compared with 470 m in patients with PVD.

## Methods

### Study design, randomisation and subjects

This randomised controlled crossover trial was conducted between October 2021 and February 2022 at the University Hospital Zurich at 470 m and in the Swiss Alps at 2500 m on the Mount Santis. Randomisation and allocation concealment were performed using software, with randomly computed block lengths. Adults diagnosed with PAH or distal CTEPH (inoperable or persistent) diagnosed according to European guidelines at the time of their last right heart catheterisation were recruited.^10 11^ Excluded were patients with unstable or concomitant severe disease, patients classified to other PH groups of PAH, if the PaO_2_ at low altitude was <7.3 kPa. Sample size was determined based on the main trial and some results of this trial were published previously.^9 12^ In the present analysis, we focused on echocardiography during IET and CWRET at high altitude versus low altitude which have not been reported. According to the randomised sequence, patients were first investigated at 2500 m, or at 470 m, with a washout period of at least 2 weeks in between. The assessments were identical. Only recordings with sufficient echocardiographic quality were included in the analysis. The study was registered on clinicaltrials.gov (NCT05107700).

### Patient and public involvement

Patients and/or the public were not involved in the design, or conduct, or reporting, or dissemination plans of this research.

### Altitude exposure

After arrival at 2500 m, participants underwent a minimum of 3 hours of rest before engaging in the IET in the afternoon on the first day. After an overnight stay, the CWRET was performed in the afternoon of the second day at high altitude.

During the altitude exposure, participants’ oxygenation and health condition were frequently monitored. When the pulse-oximetric oxygen saturation (SpO_2_) dropped <80% for >30 min or <75% for >15 min, patients were treated with supplemental oxygen for safety reasons and did perform the CWRET with supplemental oxygen, which was applied via nasal cannula.

### Echocardiography during cycling exercise

Patients performed identical IETs and CWRETs protocols at both altitudes, with the latter conducted at a constant intensity of 75% of the individual peak work-rate, both to exhaustion (Ergoselect 200 cycling ergometers Ergoline, Bitz, Germany).^9^ Echocardiography was performed at rest on the ergometer and during exercise, with measurements taken every 3 min. Echocardiographic recordings were performed using a real-time sector scanner (CX 50, Philips, Philips Respironics, Zofingen, Switzerland) with integrated colour, continuous wave (CW) and pulsed wave Doppler system. Recordings and measurements were performed according to guidelines of the American Society of Echocardiography.^13^ For the estimation of sPAP, the maximal pressure gradient of tricuspid regurgitation was calculated from maximal tricuspid regurgitation velocity (TRV) determined with CW Doppler using the modified Bernoulli equation: Δpressure=4×TRVmax^2^, without adding the right atrial pressure. CO was estimated by the Doppler velocity time integral method from the left ventricular outflow tract. TAPSE was measured in M-mode. SPAP/CO slope was calculated by (sPAP_peak_ exercise–sPAP_rest_)/(CO_peak_ exercise–CO_rest_). The TPR was calculated by sPAP/CO.

Oxygen delivery at peak exercise was calculated by (haemoglobin×arterial oxygen saturation×1.34+(arterial partial pressure for oxygen×0.003)×CO.

The co-primary outcomes were changes in sPAP and in the TAPSE/sPAP ratio during exercise between low versus high altitude.

### Data presentation and statistical analysis

To compare the co-primary outcomes of this analysis, sPAP and TAPSE/sPAP ratio at high and low altitudes were summarised as means±SE. A linear mixed model was fitted to the data with intervention (high altitude vs low altitude), period and intervention-period interaction as fixed effects, and subjects as random intercept, thus controlling for potential period effects. We did not directly test for carry-over effects between high and low altitude periods, but the model accounted for potential carry-over by including the treatment-period interaction term. This ensured that any residual effects from one period to the next were controlled for in the analysis, in accordance with the standards of crossover trials.

We tested if the intervention-period interaction could be removed from the model. Predefined time points (rest, 3 min, 6 min peak) were treated as a categorical variable to assess time effects without assuming a strictly linear trend. Model assumptions were tested by visual inspection of the homogeneity and normality of the residuals and the random effects. The covariance structure of the model was based on random intercepts for each subject, and residuals were assumed to be independent and homoscedastic. This approach is appropriate for repeated measures when there are a limited number of time points per subject, ensuring that individual variability is accounted for without modelling correlations between time points directly.

For repeated measures (different time points of echocardiographic recording during exercise), linear contrasts were defined according to the model and corrected for multiple testing by Tukey methods. The total number of contrasts tested was focused on these predefined time points.

Since some CWRET were conducted with supplemental oxygen, an interaction model was used to test whether oxygen had a statistically significant effect on the outcome variable of interest. The influence of predefined covariates on the model was tested to minimise bias. Incomplete data sets with missing values or insufficient echocardiographic quality were not imputed but were analysed using random-effects models to reduce bias, in accordance with intention-to-treat analyses.

In all analyses, a 95% CI that excluded the null effect was considered evidence for statistical significance. Analyses were performed using R-Studio software V.2024.09.0+375.

## Results

**Figure 1:**
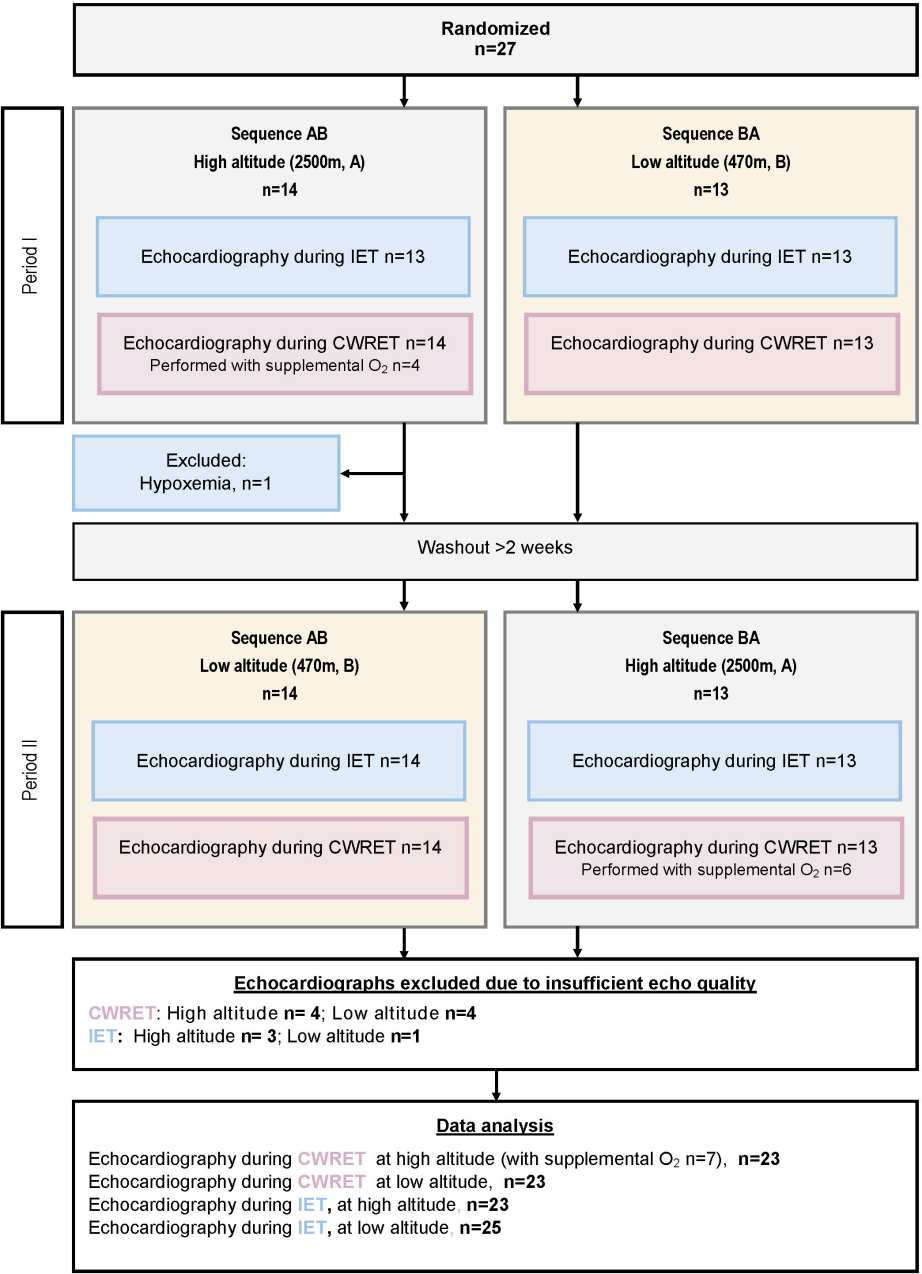
Study flow chart following the Consolidated Standards of Reporting Trials guidelines for crossover trials. CWRET, constant work-rate exercise test; IET, incremental exercise test.

A total of 27 patients (44% females, 20:7 PAH:CTEPH, age 62±14 years, 24 (89%) low risk, 3 (11%) intermediate risk) were randomised in the main trial (for baseline characteristics, see table 1).^9 12^ Out of 27 patients, one patient received oxygen after arrival at high altitude and did not perform the IET at 2500 m. After excluding recordings with insufficient echocardiographic quality, data from 25 patients during IET at low altitude, 23 patients during IET at high altitude and 23 patients during CWRET at both high and low altitudes were included in the intention-to-treat analysis (figure 1). Out of 10 patients who received oxygen at high altitude and performed the CWRET with supplemental oxygen,^12^ seven patients remained with sufficient echo quality.

**Table 1:** Baseline characteristics at low altitude

Number of participants	27
Female; male	12 (44%); 15 (56%)
Age (years)	61±13
Pulmonary arterial hypertension	20
Chronic thromboembolic pulmonary hypertension	7
New York Heart Association functional class (I–III)	I: 7 (26%), II: 19 (70%), III: 1 (4%)
ESC/ERS multipoint three-strata risk assessment	Low risk: 24 (89%), intermediate risk: 3 (11%)
Body mass index (kg/m^2^)	25.1±4.0
SpO_2_ at rest (%)	94.0±3.8
Peak oxygen uptake (mL/min/kg)	18.6±4.8
Data from last right heart catheterisation	
Mean pulmonary artery pressure (mm Hg)	42±14
Pulmonary artery wedge pressure (mm Hg)	11±4
Pulmonary vascular resistance (WU)	5.9±2.7
Cardiac output (L/min)	5.2±1.6
Cardiac index (L/min/m^2^)	2.7±0.7
PH-targeted medication	
Endothelin receptor antagonist	20 (74%)
Phosphodiesterase-5 inhibitor	11 (41%)
Intravenous prostacyclins	3 (11%)
Soluble guanylate cyclase stimulator	6 (22%)
Selexipag	3 (11%)
Calcium channel blocker	3 (11%)
Combination therapy	15 (56%)
Dual	8 (53%)
Triple	6 (40%)
Quadruple	1 (7%)

Data are presented as mean±SD, percentages or absolute numbers.

PH, pulmonary hypertension; SpO_2_, peripheral oxygen saturation.

### Echocardiography during incremental exercise test

**Figure 2:**
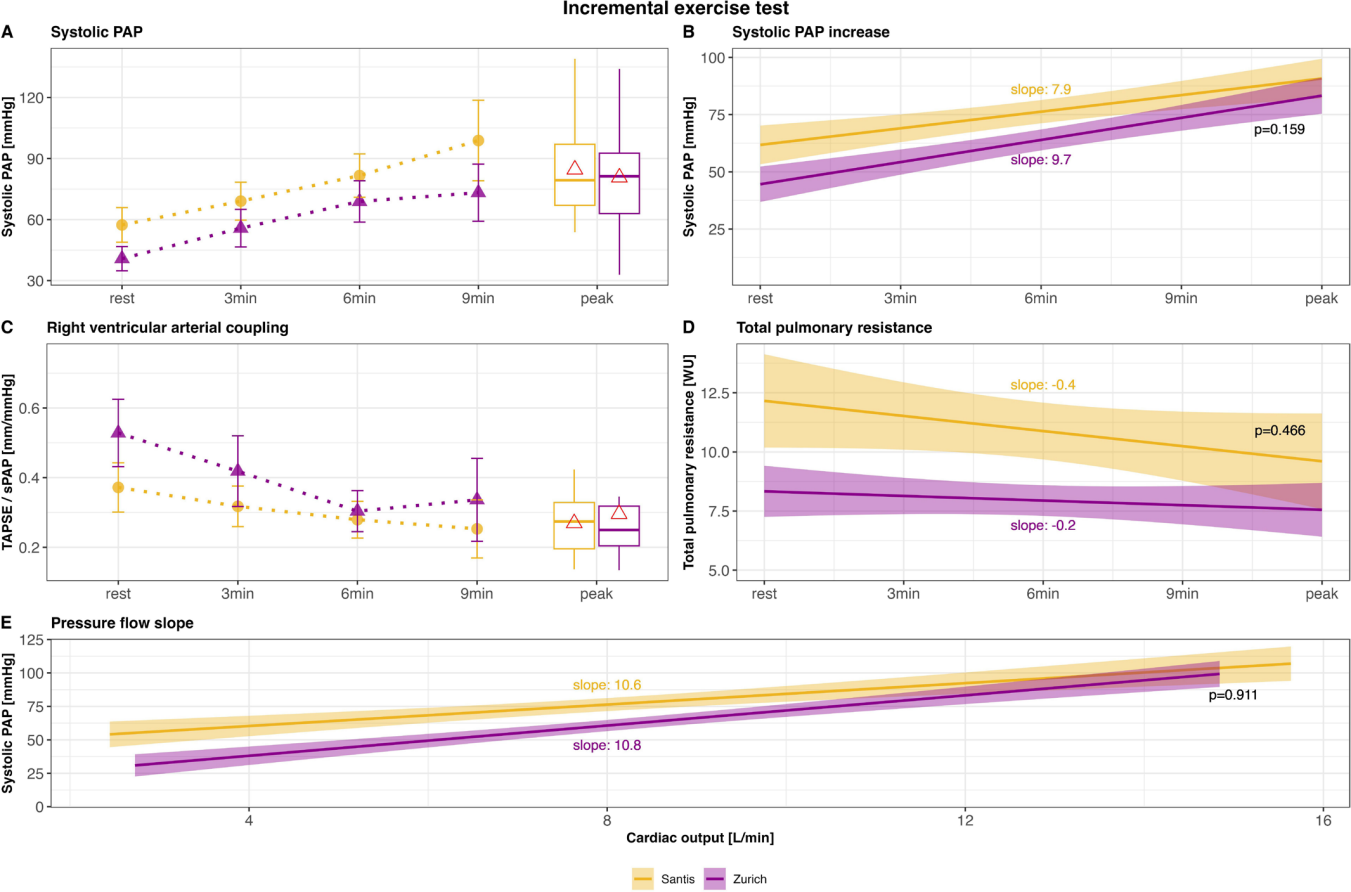
Echocardiographic data obtained during incremental exercise tests. Data are represented as mean±SE or as slopes±SE. Panels A–D depict pulmonary haemodynamics at rest (sitting on the ergometer) and during exercise. Recordings at 2500 m (Santis) are shown in yellow with circles at each time point, and recordings at 470 m (Zurich) are shown in purple with triangles at each time point. The red triangle within the box plots at peak exercise represents the mean. Peak exercise includes data from all patients, regardless of exercise duration, including those reaching peak exercise after 3 or 6 min. Panel E shows the pressure-flow slope. PAP, pulmonary arterial pressure; sPAP, systolic PAP; TAPSE, tricuspid annular plane systolic excursion.

On the first day, at rest, while seated on the ergometer, sPAP was, on average, 14 mm Hg higher at 2500 m compared with 470 m (mean difference: 14 mm Hg, 95% CI 7 to 23 mm Hg, p=0.002) (table 2, figure 2A). At both altitudes, sPAP increased linearly during exercise, with a slightly, but not statistically significantly steeper slope at 470 m (slope: 9.7 vs 7.9) compared with 2500 m (figure 2B). During incremental exercise at high altitude, sPAP remained consistently, but mostly not statistically significantly higher compared with low altitude. At peak exercise, sPAP remained unchanged between the two locations; however, exercise capacity was 11% lower at 2500 m.^9^ At rest, RV arterial coupling was, on average, 0.13 mm/mm Hg (95% CI −0.26 to −0.04 mm/mm Hg, p=0.022) lower at high altitude compared with low altitude, and successively decreased during exercise at both 2500 m and 470 m. During exercise, as well as at peak exercise, there was no statistically significant difference between the two locations (figure 2C). Heart rate during exercise was generally higher at 2500 m, while stroke volume was lower compared with 470 m. Consequently, no difference in CO or cardiac index, both of which increased linearly, was observed between the two locations (for more details, see table 2). TPR declined during exercise at both 2500 m and 470 m. However, it was statistically significantly higher at high altitude, from rest (by 3.1 WU, 95% CI 1.1 to 5.7 WU, p=0.014) to peak exercise (by 2.0 WU, 95% CI 0.6 to 3.5 WU, p=0.012) (figure 2D). The pressure flow slope was 10.8±2.1 mm Hg/L/min at 470 m and 10.6±2.1 mm Hg/L/min at 2500 m (95% CI −3.4 to 6.2, p=0.911) (figure 2E). The peripheral oxygenation (SpO_2_) by pulse oximetry at rest was 95±2% in Zurich and 90±2% at Mount Santis (95% CI −6% to −4%, p<0.001).

**Table 2:** Haemodynamics by echocardiography during incremental exercise test at low versus high altitude

	Low altitude, Zurich, 470 m N=23	High altitude Santis, 2500 m N=25	Mean change (95% CI)	P value
Rest on bike				
Systolic pulmonary arterial pressure (mm Hg)	41±4	55±4	14 (7 to 23)	**0.002**
Right ventricular arterial coupling (mm/mm Hg)	0.53±0.04	0.40±0.04	−0.13 (−0.26 to −0.04)	**0.022**
Cardiac output (L/min)	4.8±0.3	4.8±0.3	0.0 (−0.6 to 0.5)	0.884
Cardiac index (L/min/m^2^)	2.5±1.7	2.5±1.7	−0.0 (−0.3 to 0.3)	0.927
Heart rate (bpm)	73±3	82±3	9 (3 to 15)	**0.006**
Stroke volume (mL)	66±4	60±4	−6 (−14 to 1)	0.125
Total pulmonary resistance (WU)	9.3±1.3	12.4±1.3	3.1 (1.1 to 5.7)	**0.014**
SpO_2_ (%)	95±2	90±2	−5 (−6 to −4)	**<0.001**
3 min				
Systolic pulmonary arterial pressure (mm Hg)	56±5	69±5	13 (1 to 25)	**0.044**
Right ventricular arterial coupling (mm/mm Hg)	0.42±0.04	0.32±0.04	−0.10 (−0.22 to 0.02)	0.109
Cardiac output (L/min)	7.0±0.5	7.2±0.5	0.2 (−0.6 to 1.1)	0.606
Cardiac index (L/min/m^2^)	3.7±0.2	3.8±0.2	0.1 (−0.4 to 0.7)	0.637
Heart rate (bpm)	97±4	102±4	5 (1 to 10)	**0.039**
Stroke volume (mL)	73±4	71±214	−2 (−8 to 5)	0.663
Total pulmonary resistance (WU)	8.0±1.0	10.8±1.0	2.8 (0.5 to 5.0)	**0.023**
6 min				
Systolic pulmonary arterial pressure (mm Hg)	69±5	80±6	11 (−1 to 23)	0.079
Right ventricular arterial coupling (mm/mm Hg)	0.30±0.03	0.28±0.03	−0.02 (−0.09 to 0.04)	0.560
Cardiac output (L/min)	8.7±0.6	8.5±0.7	−0.2 (−1.1 to 1.4)	0.692
Cardiac index (L/min/m^2^)	4.6±0.3	4.5±0.3	0.1 (−0.8 to 0.6)	0.711
Heart rate (bpm)	115±5	120±6	5 (−4 to 15)	0.265
Stroke volume (mL)	80±5	73±5	−7 (−16 to 3)	0.154
Total pulmonary resistance (WU)	7.9±0.9	10.3±1.0	2.4 (0.2 to 4.3)	**0.012**
Peak exercise				
Systolic pulmonary arterial pressure (mm Hg)	78±26	83±6	5 (−6 to 15)	0.363
Right ventricular arterial coupling (mm/mm Hg)	0.30±0.04	0.27±0.03	−0.03 (−0.11 to 0.06)	0.523
Cardiac output (L/min)	10.3±0.8	9.2±0.7	−1.1 (−2.3 to 0.1)	0.086
Cardiac index (L/min/m^2^)	5.4±0.4	4.8±0.4	−0.6 (−1.2 to −0.0)	0.070
Heart rate (bpm)	125±6	125±6	0 (−9 to 11)	0.869
Stroke volume (mL)	85±6	75±5	−10 (−19 to −2)	**0.028**
Total pulmonary resistance (WU)	8.1±0.9	10.1±0.9	2.0 (0.6 to 3.5)	**0.012**
Pressure flow slope (mm Hg/L/min)	10.8±2.1	10.6±2.1	−0.2 (−3.4 to 6.2)	0.911
Oxygen delivery (mL/min)	2290±179	1761±147	−529 (−906 to −170)	**0.014**

Data are presented as mean±SE, mean change with the corresponding 95% CIs and p values. The end point ‘peak exercise’ refers to the individual peak values of all patients, regardless of their performance levels. Therefore, the data include values from participants who reached their peak after 3, 6 or 9 min and it might be different between high and low altitudes. Bold values indicate statistical significance.

SpO_2_, peripheral oxygen saturation.

### Echocardiography during constant work-rate exercise tests

**Figure 3:**
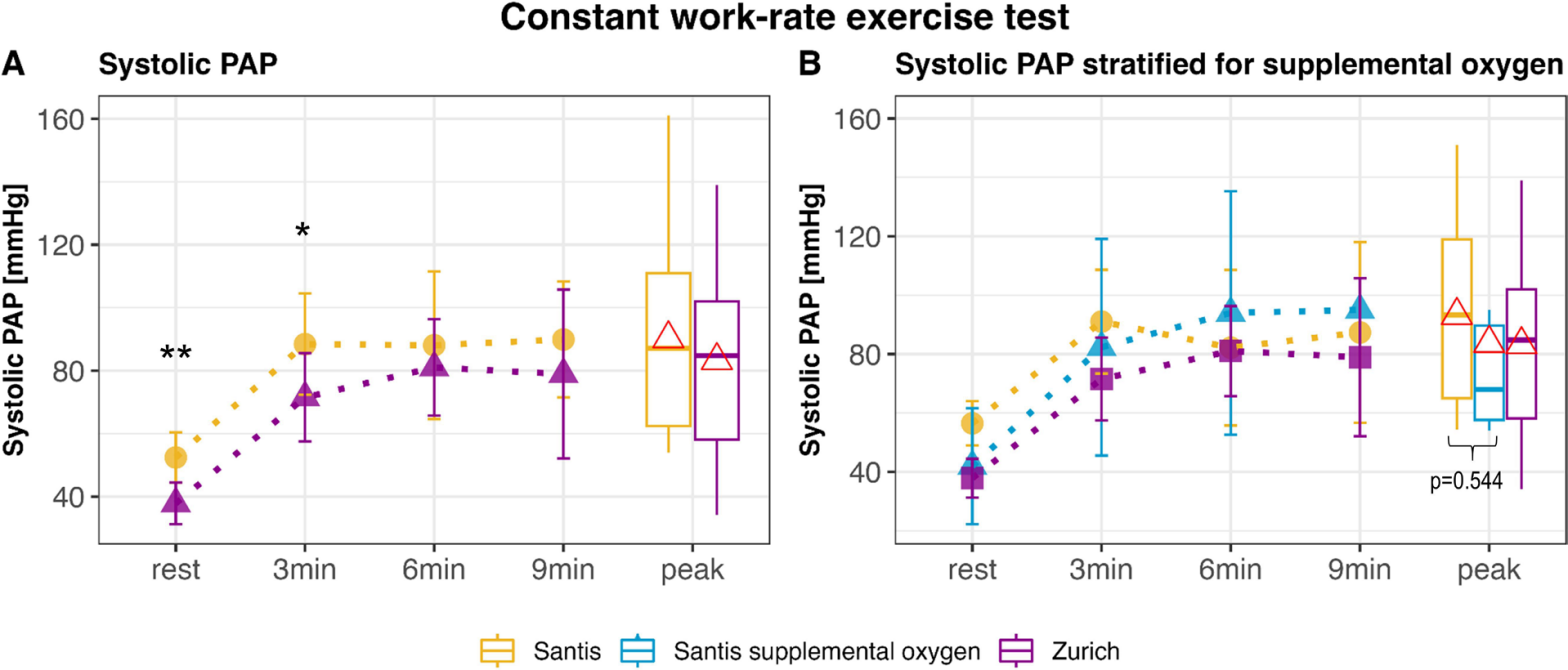
Echocardiographic data obtained during constant work-rate exercise tests. Data are represented as mean±SE. Panels A and B show the systolic pulmonary arterial pressure at rest (sitting on the ergometer) and during exercise. Panel B additionally shows the systolic pulmonary arterial pressure stratified for patients who performed the cycling test at 2500 m with supplemental oxygen. Recordings at 2500 m (Santis) are shown in yellow with circles at each time point, and recordings at 470 m (Zurich) are shown in purple with triangles at each time point. The red triangle within the box plots at peak exercise represents the mean. Peak exercise includes data from all patients, regardless of exercise duration, including those reaching peak exercise after 3 or 6 min. *P<0.05; **p<0.01. PAP, pulmonary arterial pressure.

On the second day, at rest while seated on the ergometer, sPAP remained elevated by 13 mm Hg (95% CI 5 to 23 mm Hg, p=0.004) at high altitude compared with low altitude, while RV arterial coupling was still reduced by 0.21 mm/mm Hg (95% CI −0.40 to −0.03 mm/mm Hg, p=0.032). During CWRET at 75% of peak work-rate, sPAP steeply increased during the first 3 min of exercise at both locations, followed by a plateau (figure 3A). After 3 min, sPAP was 69±8 mm Hg in Zurich and 87±7 mm Hg at Mount Santis (mean difference: 18 mm Hg, 95% CI 4 to 33 mm Hg, p=0.022). However, during the remainder of the exercise until peak exercise, there was no difference in sPAP between high and low altitudes. RV arterial coupling during exercise followed the same pattern and was slightly, but significantly, lower by −0.08 mm/mm Hg (95% CI −0.15 to −0.01 mm/mm Hg, p=0.033) at 2500 m after 3 min of exercise and remained similar to 470 m for the remainder of the exercise. TPR was also higher during CWRET at 2500 m than at 470 m, solely at peak exercise. The effect size between both locations was not statistically significantly different anymore.

The parameters at rest, including SpO_2_, as well as during exercise, did not differ statistically significantly between 2500 m and 470 m on the second day (table 3).

**Table 3:** Haemodynamics by echocardiography during constant work rate exercise tests at low versus high altitude

	Low altitude, Zurich, 470 m N=23	High altitude Santis, 2500 m N=23	Mean change (95% CI)	P value
Rest on bike				
Systolic pulmonary arterial pressure (mm Hg)	39±4	52±4	13 (5 to 23)	**0.004**
Right ventricular arterial coupling (mm/mm Hg)	0.62±0.08	0.41±0.08	−0.21 (−0.40 to −0.03)	**0.032**
Cardiac output (L/min)	4.9±0.3	5.4±0.3	0.5 (−0.1 to 1.2)	0.126
Cardiac index (L/min/m^2^)	2.6±0.2	2.9±0.2	0.3 (−0.1 to 0.6)	0.142
Heart rate (bpm)	82±3	84±3	2 (−3 to 7)	0.406
Stroke volume (mL)	60±4	64±4	4 (−2 to 9)	0.188
Total pulmonary resistance (WU)	8.3±0.9	10.6±0.9	2.3 (0.0 to 4.6)	0.054
SpO_2_ (%)	95±2	93±3	−2 (−4 to 0)	0.062
3 min				
Systolic pulmonary arterial pressure (mm Hg)	69±8	87±7	18 (4 to 33)	**0.022**
Right ventricular arterial coupling (mm/mm Hg)	0.38±0.04	0.30±0.04	−0.08 (−0.15 to −0.01)	**0.033**
Cardiac output (L/min)	10.5±0.8	9.7±0.8	−0.8 (−2.6 to 0.9)	0.405
Cardiac index (L/min/m^2^)	5.7±0.4	5.1±0.4	−0.6 (−1.5 to 0.4)	0.271
Heart rate (bpm)	128±5	130±5	2 (−6 to 10)	0.576
Stroke volume (mL)	82±6	76±6	−6 (−17 to 6)	0.371
Total pulmonary resistance (WU)	7.0±1.1	9.9±1.2	2.9 (0.4 to 5.4)	**0.039**
6 min				
Systolic pulmonary arterial pressure (mm Hg)	79±8	88±9	9 (−3 to 21)	0.162
Right ventricular arterial coupling (mm/mm Hg)	0.31±0.04	0.31±0.05	0.00 (−0.07 to 0.08)	0.998
Cardiac output (L/min)	11.7±1.0	12.2±1.0	0.5 (−0.5 to 1.5)	0.308
Cardiac index (L/min/m^2^)	6.3±0.5	6.5±0.5	0.2 (−0.3 to 0.8)	0.383
Heart rate (bpm)	138±5	143±5	5 (0 to 10)	0.085
Stroke volume (mL)	86±7	87±7	1 (−5 to 7)	0.712
Total pulmonary resistance (WU)	7.0±0.9	8.3±1.0	1.3 (0.2 to 2.3)	**0.047**
Peak exercise				
Systolic pulmonary arterial pressure (mm Hg)	81±7	88±7	7 (−5 to 17)	0.294
Right ventricular arterial coupling (mm/mm Hg)	0.34±0.04	0.29±0.04	−0.05 (−0.14 to 0.04)	0.239
Cardiac output (L/min)	11.8±0.9	10.5±0.9	−1.3 (−3.0 to 3.5)	0.140
Cardiac index (L/min/m^2^)	6.4±0.4	5.6±0.4	−0.8 (−1.9 to 0.1)	0.102
Heart rate (bpm)	138±5	135±5	−3 (−9 to 3)	0.317
Stroke volume (mL)	86±6	79±6	−7 (−18 to 4)	0.212
Total pulmonary resistance (WU)	7.3±1.0	9.1±1.0	1.8 (−0.2 to 3.9)	0.092
Oxygen delivery (mL/min)	2318±86	1881±644	−440 (−440 to −433)	**<0.001**

Data are presented as mean±SE, mean change with the corresponding 95% CIs and p values. The end point ‘peak exercise’ refers to the individual peak values of all patients, regardless of their performance levels. Therefore, the data include values from participants who reached their peak after 3, 6 or 9 min and it might be different between high and low altitudes. Bold values indicate statistical significance.

SpO_2_, peripheral oxygen saturation.

#### Oxygen therapy during CWRET

In seven patients who received oxygen during CWRET at high altitude due to predefined safety criteria, there was no statistically significant difference in right heart parameters compared with those without oxygen therapy. At rest, oxygen reduced sPAP so that it was no longer significantly different from sPAP at rest at low altitude (figure 3B).

### Oxygen delivery

At peak exercise, oxygen delivery was lower at high altitude compared with low altitude by −529 mL/min (95% CI −906 to −170 mL/min, p=0.014) in IET and −440 mL/min (95% CI −440 to −433 mL/min, p<0.001) in CWRET.

## Discussion

The present trial in patients with PVD shows that pulmonary haemodynamic changes during exercise were similar at 2500 m compared with 470 m, even though absolute levels of sPAP, HR and CO were higher at high altitude, whereas RV arterial coupling was lower. The patterns of exercise haemodynamics were strongly influenced by the exercise protocol, independent of altitude exposure.

Healthy individuals and patients with PVD reveal an elevated sPAP at rest and at end-exercise at high altitude. In healthy individuals, resting PAP gradually declines over time due to acclimatisation effects.^14^,^17^ In the present study during IET performed after approximately 4 hours at 2500 m, sPAP increased linearly with identical slopes at both high and low altitudes. Thus, this linear rise of around 8–10 mm Hg/min was independent of altitude exposure, starting from a higher baseline value at high altitude (figure 2B). At peak exercise, sPAP, HR and CO were identical, even though participants had 11% higher IET exercise capacity at 470 m.^18^ Therefore, it is likely that sPAP during exertion contributes to exercise limitation in patients with PVD, as known for the HR and the CO in general. TAPSE/sPAP as a surrogate for the RV arterial coupling was also similar at peak exercise at both altitudes. When the RV is unable to successfully maintain its coupling during exercise, the pressure response cannot be effectively executed and the patients stop cycling. Grünig *et al* described this RV dynamic adaptation to increased afterload during exercise as RV contractile reserve, which was investigated in 124 patients with PVD by echocardiography and found to be of highly prognostic value.^19^ The RV arterial coupling was higher at rest at low compared with high altitude, but unchanged during exercise until peak exercise. From rest to peak exercise, the RV arterial coupling constantly declined at both altitudes (figure 2C). It shows an opposite pattern to sPAP, and the RV contractile reserve decreases with increasing afterload during progressive exercise, independently from altitude exposure. Interestingly, Ulrich *et al* investigated exercise haemodynamics in 52 highlanders and 22 lowlanders, who did not suffer from apparent diseases. They found that neither group at its respective living altitude showed a decrease in RV arterial coupling during exercise.^20^

The TPR was significantly higher at rest and during exercise at 2500 m compared with 470 m, which can be explained by a greater increase in PAP relative to CO. However, CO measures during exercise are challenging, which indicates that these measures have to be interpreted with caution. During exercise, at both locations, the TPR slightly decreased with increased CO, due to recruitment and/or distension of pulmonary vessels.^2 21^ This was also shown with simulated hypoxia in 28 patients with PVD examined by exercise echocardiography.^22^ The relationship of sPAP and CO during exercise is assumed to follow a linear relationship in patients with PVD and is expressed by the sPAP/CO slope, which was similar at high compared with low altitude.^1^ At low altitude, a sPAP/CO slope >3 mm Hg/L/min is a sign of exercise PH and in patients with PVD, a higher slope is associated with poorer survival.^8 10 23^

The increase in sPAP during CWRET showed a different pattern. SPAP rose steeply during the first 3 min, independently of altitude exposure, followed by a plateau once patients reached a steady state, remaining constant until peak exercise (figure 3A). This shows that the rise in PAP is strongly dependent on the exercise protocol. Since exercise pulmonary haemodynamics during right heart catheterisation are recommended to be measured using incremental ramp or step protocols, assuming linearity of the sPAP/CO slope may be disputable when using the latter.^24^ Applying a multipoint slope method, as introduced by previous studies, may be a reasonable solution here.^8 25^ Due to predefined safety criteria, some patients received oxygen therapy during the night and performed the CWRET, which was performed in the afternoon of the second day at 2500 m with 3 L supplemental oxygen, applied via nasal cannula.^12^ At rest, while patients were seated on the ergometer and breathing calmly, oxygen therapy reduced sPAP to values closely to those at low altitude. However, no statistically significant difference was identified between tests with and without oxygen therapy when patients were cycling at high altitude. Potential explanations could be that the oxygen flow was too low or that patients also breathed through their mouths, especially during exercise. The flow rate was chosen to align with usual care, meaning that most patient with prescribed ambulatory oxygen by portable devices had flow rates of around 3 L/min. When applying high flow oxygen therapy, we could have expected the sPAP to decline.^26^

In both IET and CWRET, oxygen delivery at peak exercise was significantly lower at high compared with low altitude. Since exercise capacity at 470 m was also higher compared with 2500 m, it would have been interesting to investigate oxygen delivery at an identical time point during exercise at high versus low altitude. However, we only collected arterial blood samples at rest and peak exercise. At peak exercise (IET and CWRET), sPAP values appeared to converge across participants, suggesting a possible ceiling effect, where further increases in sPAP may be limited by the RV contractile reserve, as described in PVD at low altitude by Grünig *et al*.^19^ This may also be supported by the RV-arterial coupling during exercise, where the differences became smaller with increasing exercise intensity.

We included only stable patients who had not modified their PVD-targeted therapy for at least 4 weeks prior to the study. Additionally, participants were instructed to take their medication as they normally would at home. Therefore, we do not expect the timing of medication intake to have significantly influenced our findings, given that both tests at high and low altitude were conducted at similar times of day.

Our findings may have important implications for air travel in patients with PVD, as commercial airline cabins are typically pressurised to altitudes of approximately 1800–2400 m. While our study was conducted at 2500 m, the observed haemodynamic responses at rest suggest that even moderate hypobaric hypoxia impacts sPAP and RV function, although the changes during exercise are similar. This highlights the need for individualised risk assessment and potential preflight evaluations, particularly in patients with more advanced disease.

### Limitations

The sample size in this trial was rather small; however, the crossover design without inter-rater variability allows us to reduce the sample size drastically without a loss in statistical power and is therefore the method of choice for rare diseases like PVD.^27^ There was a loss of data due to insufficient echocardiographic quality. However, we decided to include only high-quality images to preserve the interpretability of our data. Due to predefined safety criteria, a small group of patients performed the CWRET with supplemental oxygen therapy.^12^ This reduces the sample size for the main analysis, and the oxygen sample itself may be too small to detect a potential effect of oxygen therapy on pulmonary haemodynamics during CWRET.

### Where to go?

Although overall haemodynamic levels are influenced by high-altitude exposure, the increase in sPAP, decrease in RV-arterial coupling, reduction in TPR and pressure-flow slope during exercise appear to be independent of altitude exposure, but starting from different baseline values. This offers interesting clinical applications. These findings highlight that pulmonary haemodynamic responses during exercise, rather than resting values alone, may provide important prognostic insights for patients with PVD who wish to travel to and be physically active at altitude. Assessing these parameters at low altitude could help identify individuals at higher risk of clinical worsening at high altitude, guiding personalised management strategies and precautions for both patients and caregivers. Further research should therefore focus on studying parameters at low altitude to predict clinical worsening and events at high altitude. Furthermore, data on long-term exposure to high altitude are needed to support decision-making and provide recommendations for patients with PVD who wish to travel to and be active at high altitude.

## Conclusion

This study shows that predominantly low-risk patients with PVD revealed a similar increase in sPAP, decrease in RV arterial coupling and pressure-flow slope during exercise at 2500 vs 470 m despite starting from different baselines, which points against a hypoxia-associated harm to the cardiopulmonary system during exercise, at least on short-term exposure. The patterns of these exercise pulmonary haemodynamics were strongly dependent on the exercise protocol. Further research should focus on low-altitude predictors of clinical worsening and events at high altitude in patients with PVD.

## Data availability statement

Data are available on reasonable request.
